# COVID-19, sex, and gender in China: a scoping review

**DOI:** 10.1186/s12992-022-00804-w

**Published:** 2022-02-04

**Authors:** Huiyun Feng, Connie Cai Ru Gan, Diego Leiva, Bao Ling Zhang, Sara E. Davies

**Affiliations:** 1 School of Government and International Relations, Griffith University, Nathan, Brisbane, QLD Australia; 2 Centre for Environment and Population Health, Griffith University, Qld Nathan, Australia; 3 Institute for Hospital Management, Tsinghua University, Shenzhen, China

**Keywords:** Sex-disaggregated data, China, COVID-19, Gendered impacts

## Abstract

**Background:**

During the course of the COVID-19 pandemic, states were called upon by the World Health Organization to introduce and prioritise the collection of sex-disaggregated data. The collection of sex-disaggregated data on COVID-19 testing, infection rates, hospital admissions, and deaths, when available, has informed our understanding of the biology of the infectious disease. The collection of sex-disaggregated data should also better inform our understanding of the gendered impacts that contribute to risk of exposure to COVID-19. In China, the country with the longest history of fighting the COVID-19 infection, what research was available on the gender-differential impacts of COVID-19 in the first 6 months of the COVID-19 pandemic?

**Methods:**

In this scoping review, we examine the first 6 months (January–June 2020) of peer-reviewed publications (*n* = 451) on sex and gender experiences related to COVID-19 in China. We conducted an exhaustive search of published Chinese and English language research papers on COVID-19 in mainland China. We used a COVID-19 Gender Matrix informed by the JPHIEGO gender analysis toolkit to examine and illuminate research into the gendered impacts of COVID-19 within China.

**Results:**

In China, only a small portion of the COVID-19-related research focused on gender experiences and differences. Near the end of the six-month literature review period, a small number of research items emerged on women healthcare workers, women’s mental health, and pregnant women’s access to care. There was an absence of research on the gendered impact of COVID-19 amongst populations. There was minimal consideration of the economic, social and security factors, including gender stereotypes and expectations, that affected different populations’ experiences of infection, treatment, and lockdown during the period of review.

**Conclusion:**

At the outset of health emergencies in China, gender research needs to be prioritised during the first stage of an outbreak to assist with evaluation of the most effective public health measures, identifying access to healthcare and social welfare barriers amongst priority communities. Gender stereotypes and gendered differences lead to different patterns of exposure and treatment. The exclusion of this knowledge in real time affects the design of effective prevention and recovery.

**Supplementary Information:**

The online version contains supplementary material available at 10.1186/s12992-022-00804-w.

## Background

### Tracking sex and gender in real time of COVID-19

The collection of sex-disaggregated health data has been a strong recommendation of the Inter-Agency and Expert Group on Sustainable Development Goal Indicators (IAEG-SDGs) and the World Health Organization (WHO) National Action Plan for Health Security [1]. The 2019 Novel Coronavirus Strategic Preparedness and Response Plan, published by the WHO on 4 February 2020, emphasised: “Disaggregated data on age, sex, pregnancy status and outcome (as appropriate) should be reported” [2]. However, as reported by Global Health 5050, “no single country is reporting sex disaggregated data across the key indicators that show who is getting tested, sick and dying from COVID-19.” This means that we do not know “the sex of roughly 4 in 10 cases and 3 in 10 deaths globally” [3].

The collection of sex-disaggregated data informs real-time understanding of the biology of an infectious disease as well as the social and economic factors that contribute to risk of exposure [4, 5]. For the ongoing COVID-19 pandemic, the absence of sex-disaggregated data remains an information black hole [6]: Is the biological risk of infection the same for women and men? Are more women getting tested than men? Are women observing social distancing protocols more than men? Research has shown that sex-disaggregated data from testing to fatalities improves the targeting of risk communication, sentinel surveillance, and treatments [4]. For example, sex-disaggregated data can reveal important data about the COVID-19 clinical pathway: who is turning up for testing, who is requiring hospitalisation, and who is dying in higher numbers.

For more empirical and policy relevant research, a gender analysis of health emergencies has the “potential to offer new perspectives, pose new questions and, importantly, enhance social equalities by ensuring that research findings are applicable across the whole of society” [7]. Sex refers to the biological attributes that distinguish organisms as male, female, intersex and hermaphrodite (ibid). Gender refers to psychological, social and cultural factors that shape attitudes, behaviours, stereotypes, technologies and knowledge (ibid). It is vital to understand how gender norms are expressed during crisis because “they play a role in shaping women and men’s (often unequal) access to resources and freedoms, thus affecting their voice, power and sense of self” [8].

Understanding the local gendered effects of COVID-19 in real time requires attention to equal participation and representation during the health emergency response [8, 9]. Local risk communication and information may have heteronormative gender norms that affect how individuals view their responsibility to manage risk individually and as carers, i.e. who needs to provide home schooling, who needs to work, who needs to go shopping. Gender inequalities may determine an individual’s access to health services and this knowledge can inform social policy which can, in turn, complement public health measures to ensure that populations are not taking on additional health risks due to unequal economic and social burdens during lockdown periods [10].

China was the first country to report the outbreak of the virus on 31 December 2019; and it was one of the first countries to recover from the first wave of the virus. In March 2020, the Asia Pacific Gender in Humanitarian Action Working Group recommended that all states in the Asia Pacific needed to prioritise the collection of disaggregated data related to the outbreak by “sex, age, and disability”; and this data needed to be analysed in order to “understand the gendered differences in exposure and treatment and to design differential preventive measures” [11].

In December 2019, the Chinese government had just ratified and adopted the Basic Healthcare and Health Promotion Law, which guaranteed Chinese citizens’ equal access to basic health care services, including a particular emphasis on child and maternal health. Prior to the outbreak of COVID-19, the National Health Commission had been pursuing a range of improvements to its collection and analysis of health data, including the introduction of national level sex-disaggregated data. Over the last 10 years, the Chinese government has progressed a range of gender equality laws in the workplace and recently introduced an anti-domestic violence law. However, in practice, social attitudes to gender equality and women’s empowerment have been described as requiring more awareness. In a survey of men’s and women’s attitudes on vulnerability to climate change and disaster risks, it was found that women have “less decision-making power on issues other than daily expenses” and “women have fewer opportunities to participate in their communities’ public affairs” [12]. Moreover, the representation of women in national and sub-national discussions on disaster risk and reduction tends to position women as “vulnerable” rather than “active agents” [13]. The UN, in its 2019 *China Annual Report* also noted the need to “strengthen the evidence base and understanding of China’s progress on international, regional and national commitments towards gender equality and women’s empowerment” [14]. In the same report, UN Women documented ongoing work to “tackle social norms and stereotypes that sustain gender inequality and trigger gender based violence” [15].

Any emergency, including health emergencies, give rise to gendered experiences of infection, illness, vulnerability, and recovery [4]. Gendered experiences overlap with age, income, ethnicity, and disability. These experiences and differences interact to create differentiated risks of exposure amongst populations. Gender analysis, which includes the analysis of overlapping themes like social, economic, and physical security, pre-existing health conditions and access to care, help public health officials identify and understand how different populations will interpret and respond to public health measures [6]. Sex, age and disability disaggregated data, on their own will not reveal the social norms and stereotypes that dictate how different populations respond to public health measures and lockdown orders. A health emergency places stress on pre-existing inequalities and discrimination. As such, a health emergency can initiate and exacerbate inequality and discrimination [9]. Emergency response measures, while necessary, can place different groups into situations that are dangerous or harmful. Rapid research into gender, as well as ethnicity, disability, age, and economic experiences during health emergencies is vital to identify the healthcare measures, social supports, and protections needed to ensure public health compliance and equitable recovery. This paper examines the real-time research that was being conducted on COVID-19, sex, and gender during China’s first wave of infections.

## Methods

A scoping review method was adopted to examine the volume, variety and nature of the evidence on COVID-19, sex and gender in China. The benefit of a scoping review is that it is useful to identify gaps in the rapid research published that is intended to assist the planning of public health response and provide directions for commissioning future research [16]. Our interest was in ascertaining whether the February 2020 call for states to provide sex-disaggregated data also facilitated a growth in gender analysis of the COVID-19 pandemic in locations first affected such as China. The scoping review examined the first 6 months (January–June 2020) of peer-reviewed publications on the primary (COVID-19 infection and illness) and secondary effects (social welfare and security) of COVID-19 on sex and gender in China, the country with the longest history of fighting the COVID-19 infection.

Global and local research publication sources were searched using context-sensitive search terms and a combined electronic and manual search was conducted to identify primary studies for this scoping review. Eligible peer-reviewed literature was identified through Web of Science and Google Scholar (for English language literature); CNKI, WanFang, Weipu and Google Scholar (for Chinese literature). The search terms covered all areas, including Medical Subject Headings (MeSH) terms, subject headings and keywords. The search strategy in the proposed databases was based on the search syntax published from 31 December 2019 to 30 June 2020 (see Supplement A).^1^

The screening and study selection phase involved several steps. First, two reviewer coders independently screened titles and abstracts to determine the inclusion status. To qualify for the review, studies had to describe the effects of COVID-19 (or SARS-Cov-2) with reference to one or more of the following keywords: male/men or female/women or gender. Second, full text of any items with potential to meet the review inclusion criteria was obtained and assessed against the review inclusion criteria by the same two coders (see Table 1). Non research articles were excluded, such as editorials, commentaries, reviews, book chapters, news and blogposts. Discordant views were resolved by consensus or by reference to a third coder.

**Table 1:** Selection of studies on inclusion and exclusion criteria

	Inclusion criteria	Exclusion criteria
**Publication types**	Peer-reviewed, empirical studies	Editorials, commentaries, review, Social media posts, news articles, press release, blogposts, conference proceedings, dissertations), book chapter, blog post
**Quality/ Evidence**	Publications were categorised into core and non-core journals according to Peking University List of Core Journal 2018 (version 8)	
**Language**	English or Simplified Chinese^b^	Full text not in either English or Chinese
**Database/ Search Engine**	English literature: Web of Science, Google Scholar Chinese literature: CKNI, WanFang, Weipu, Google Scholar site: .gov.cn; site: .org	
**Published date**	^a^December 31st 2019 to June 30th 2020	Online first (without full text)
**Study Population**	Mainland China	Hong Kong, Macau, Taiwan and overseas Chinese

^a^First case reported to the WHO Country Office in China

^b^Simplified Chinese is the official written Chinese language. There are two standard character sets of Chinese written language: Simplified and Traditional. Simplified Chinese characters are used in Mainland China, which is the scope of this study

The design of the scoping review protocol used was derived from the guidance published by Arksey and O’Malley [17] and data extraction tools (including data extraction sheets for English and Chinese studies that were developed and adapted to the review question). Data on study settings, participants, methods of data collection and findings were extracted from the included studies by one author and checked by another. Separate data extraction sheets were developed and piloted for both languages. Disagreements on classification and thematic coding were resolved by group discussions.

The data analysis applied both quantitative (i.e. frequencies and percentages) and qualitative (i.e. thematic analysis) methods. The authors analysed each research publication to establish the degree to which sex and gender-related considerations or both were researched when compiling an understanding of the pandemic (in real time).

The final sample includes studies covering a variety of study designs: descriptive epidemiological studies, clinical characteristic and interventional studies (eg. diagnosis, treatment, before and after) as primary effect studies; studies examining the secondary impact of SARS-CoV-2 on one or more sexes; as well as studies that researched the secondary impact of SARS-Cov-2 (e.g. movement restriction) as part of an intervention to change some aspect of policy or practice. The retrieved studies were categorised under five themes identified in a rapid gender assessment matrix designed for the COVID-19 pandemic [15]. The five areas selected in the matrix aim to identify what knowledge, experiences and responses to the COVID-19 outbreak were informed by risk (of exposure), illness, and access to health services, as well as social, economic, and security conditions. The gender and health matrix methodology organises research into five categories that seek to uncover where gender research is concentrated and/or missing: individual risk and vulnerability, experience of illness and treatment, general access to health services, social impacts of crisis, and security impacts of crisis. The matrix, as an analytical tool, permits examination of concentration of gendered investigation and studies during an event, such as the COVID-19 pandemic, and serves to highlight knowledge gaps. Below, for each category we describe the general findings from the assignment of articles under each theme [18].

The vast literature was grouped, analytically, under the five themes through quantifiable means. The authors frequently collaborated to ensure shared understanding of the thematic areas to analyse and group each publication. The authors do not claim to comment on the quality of the studies, nor evaluate the strength of the evidence or data presented in the publications. A limitation of this research is that the included studies did not examine men, transgender, or non-binary gendered experiences.

## Results

**Fig. 1:**
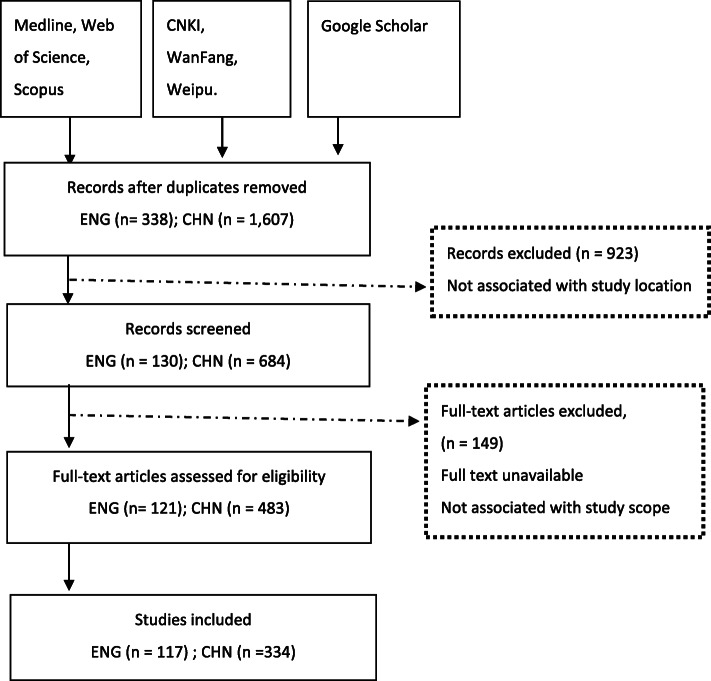
PRISMA flow diagram

From the 2,083 articles initially identified, 451 empirical studies met the inclusion criteria (see Table 1). The eligible studies are reported in the PRISMA flow diagram (Fig. 1) and their main characteristics are outlined in Table 2. Out of the 451 studies included in this review, 74% (*n* = 334) were published in Chinese and 26% (*n* = 117) were in English. The majority of articles were published in April 2020, with most of the research conducted in Hubei, followed by Sichuan and Guangdong.

**Table 2:** Description of included studies

Variable	English	Chinese
Total – *N*	117	334
Study Published date		
2020 (Did not specify month)	4	10
Jan	1	–
Feb	7	46
March	23	79
April	36	100
May	33	66
June	9	33
Sample size – median (range)	149 (1–82,858)	84 (1–44,038)
Study design		
Case study	11	190
Cross-sectional	26	52
Controlled before-after	1	12
Cohort study (retro)	65	
Data collection methods		
Survey	29	83
Medical record	81	319
Interview	0	4
Document / Surveillance Data	11	227

**Fig. 2:**
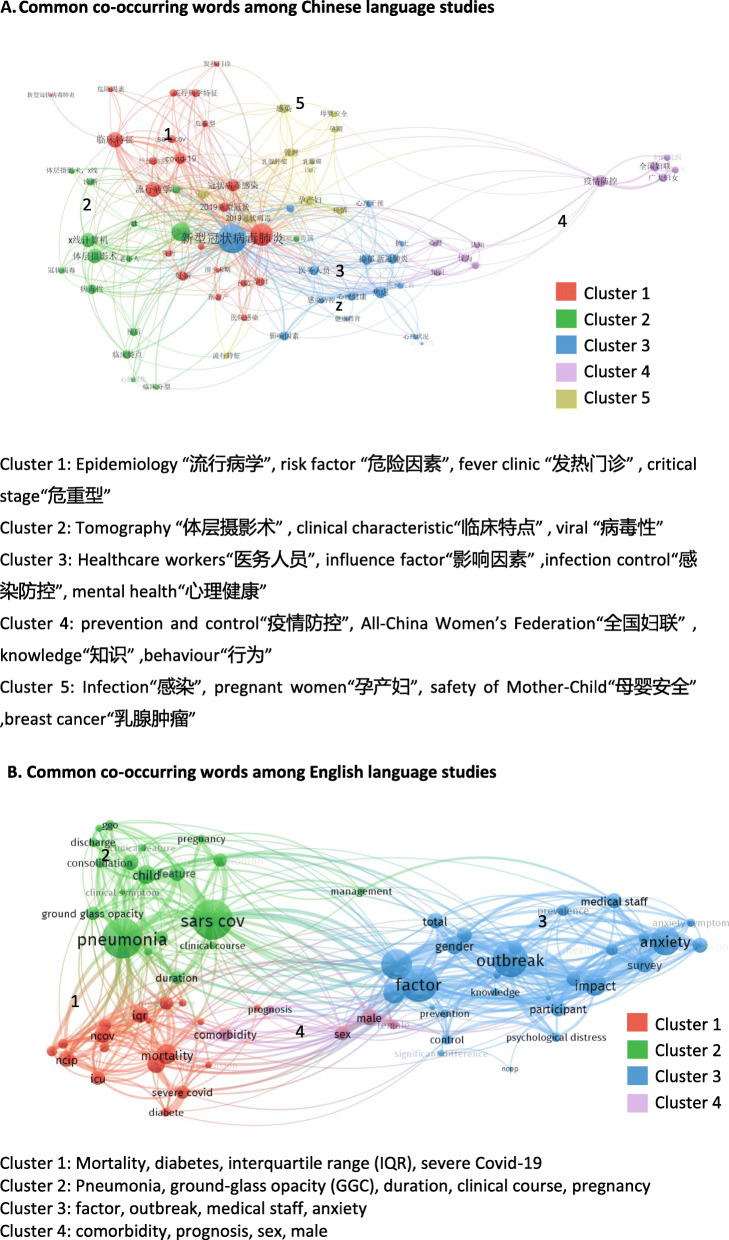
**A** Common co-occurring words among Chinese language studies. **B** Common co-occurring words among English language studies

The co-occurrence of key terms from retrieved studies highlights the primary focus of the publications and denotes research topics (Fig. 2A and B). Some of the co-occurrence key terms are related to epidemiological characteristics including outbreak, male, female, mortality, diabetes and medical staff. Others are related to clinical diagnosis such as duration, anxiety, computer tomography and fever clinic. The Chinese and English language research papers included sex as a variable but the papers, on the whole, failed to analyse the everyday social aspects of gender and its relationship to COVID-19 infection, recovery and death.

The goal of the scoping review is to understand the extent of rapid research that engaged with the gender-related considerations identified in the Gender Analysis and COVID-19 Matrix during the first wave of COVID-19 in China. The majority of studies (both English and Chinese papers) focused on sex differences to understand the impact of the pandemic. The review reveals very little “real time” research and consideration of whether gender, as well as disability, income, ethnicity and age, contributed to exposure, infection, and recovery [18, 19].

We identify two explanations for this knowledge gap. The first is that real-time research on health emergencies and their gendered effects has been identified as an analytical gap across most countries and, in turn, this has affected gender-inclusive health policy response and recovery [6, 18]. The second explanation, based on findings from this scoping review, is that research in China tends to conflate sex and gender. Research on sex-disaggregated data is presented as findings on gender, i.e. women healthcare workers experienced more stress during COVID-19, rather than analysing whether there were gendered inequalities and differences in roles and expectations that distinguished the experiences of women healthcare workers from those of their male counterparts.

We can establish from this scoping review that, conceptually, real-time sex and gender analysis was prioritised when it intersected with health impacts and healthcare work considerations. There was little real-time published research available on the gendered impacts of COVID-19 on Chinese society in the first 6 months in China.

As detailed below, the review finds that across each of the five matrix categories, individual risk and vulnerability, experience of illness and treatment, general access to health services, social impacts of crisis, and security impacts of crisis, the research articles include sex- disaggregated data but analytically assume traditional gendered roles/activities. There are few examples of published articles that analyse the COVID-19 experience in China with the intention of understanding the “gendered differences in exposure and treatment and to design differential preventive measures” [11].

### Risk and vulnerability

For this category, research is included if the study identifies individual or group risk and vulnerability of infection. We included articles identifying individual high-risk of COVID-19 infection, including pregnant persons, gender-specific conditions such as breast cancer, cervical cancer, uterine cancer, ovarian cancer, prostate cancer, neurotypical, congenital, birth trauma, genetic and/or hereditary conditions, age (elderly, youth, child, infant), and autoimmune conditions (HIV, SLE, NLR. AFL). In the first stage of the outbreak, the majority of sex-disaggregated studies on infection (Chinese *n* = 22; English *n* = 7) detailed the clinical characteristics of COVID-19 patients with flu or fever symptoms and an association with pre-existing conditions such as breast cancer [20], cardio vascular diseases [21], pneumonia and diabetes [22]. The source of these studies were medical records, surveillance data, or a combination of both. Overall, the risk of COVID-19 infection to men is presented as higher than women, but certainty of sex-disaggregated results was uneven due to inconsistent participant recruitment and diagnostic methods.

Half of all published articles identified in this review examined sex-disaggregated risk and vulnerability to SARS-Cov-2 (*n* = 235), but only one study examined whether gendered roles could be associated with risk of infection. The majority of research consisted of sex-disaggregated studies (Chinese and English) on the risk of COVID-19 to medical staff (*n* = 82), the elderly (*n* = 29), children and infants (*n* = 34), pregnant women (*n* = 18) and others (*n* = 14). Publications on the first wave of the outbreak examined the impact of COVID-19 pandemic on women healthcare workers and carers in Hubei [23]. Women healthcare workers are the majority of healthcare workers across China, including Hubei. Some studies examined the prevalence of PPE-related skin injuries among nurses [24]. Several research papers were published on women healthcare workers’ struggle to work under the measures taken by the authorities [25]. There was a noticeable increase in the number of studies on healthcare workers’ mental health assessed through online surveys focused on sleep quality, anxiety and stress [26, 27]. These studies tended to focus on women healthcare workers. There was little examination of whether this high volume of women healthcare workers experiencing stress and precarity is due to social gender norms that associate women with vulnerability (i.e. it is more socially acceptable for women to discuss personal vulnerabilities than men). There is also the fact that women healthcare workers were primarily the frontline staff, who were at high potential risk of infection due to the illness’ characteristics of high transmission efficiency, rapid deterioration and pathogenicity. We note there is an absence of men and their experiences as healthcare workers at risk of infection. The multitude of papers that refer to women healthcare workers and mental illness, with lack of consideration as to whether and if men were equally affected, points to unchallenged gender stereotypes in the research publications examining the impact of COVID-19 on risk of infection and risk of associated illness.

An additional 29 studies (Chinese and English) researched the transmission mode of COVID-19: vertical transmission, nosocomial transmission, organ donation and through family cluster [28–31]. Of these studies that analysed the sex-disaggregation only one study did so with a gender lens concluding that women’s uptake in care roles, higher frequency of hospital visits and household chores, makes them more vulnerable to infection [31].

### Illness and treatment

The articles in this category focussed on the clinical observations and epidemiological studies on COVID-19 including fatality rates, testing and treatment. As government policies encouraged exploring alternative treatments for COVID-19 (eg. Guangdong, Shaanxi Provincial government COVID-19 TCM treatment pilot plan), we observed a number of research papers that integrated Traditional Chinese Medicine and other alternative and complementary medicines [24, 32–38]. These studies presented no research into significant [34] sex differences in terms of diagnostic of COVID-19 confirmed cases.

There were 91 articles discussing the detection of SARS-Cov-2 through stool, gastrointestinal tract, saliva, and urine samples examined using radiology methods or laboratory that were sex-disaggregated [39, 40]. In one study, researchers found that SARS-CoV-2 can be present in the semen of patients with COVID-19, and SARS-CoV-2 may still be detected in the semen of recovering patients [41]. There were no studies, that we could find, on gendered practices in seeking COVID-19-related care and recovery. The only exception near the end of the six- month period was studies that recruited research participants who were pregnant [42–45]: 53 articles were published in this period discussing COVID-19 treatment in conjunction with pregnancy.

### Access to health services

For this category we included articles that analysed who sought access to health services during China’s first Covid-wave lockdown. Specifically, they encompass those who sought COVID-19 testing, as well as the health, social or/and psychological supports available to populations during the first wave. The scale of China’s lockdown during this period was immense: in Wuhan alone 11 million people were in lockdown during the period of scoping review. There were, however, no published papers examining the reorganisation of existing care services and treatments to meet specific sexual and reproductive healthcare needs, the readjustment of care and support for those with mental illness, the specific healthcare needs of those with disabilities, or aged care.

During this period, most articles examined healthcare service management during the outbreak for, specifically, antenatal care planning [46, 47], patient triage based on the risk level, admission control and measures on counteracting emergencies, and designating safe zones for non-Covid-19 patients [47–49]. There were a small number of publications on the need to ensure virtual healthcare where possible, and healthcare providers were encouraged to expand their remote care practices [50–52]. For example, 233 out of 294 midwifery clinics in Guangdong province provided a COVID-19 hot-line service, and 186 clinics delivered telehealth services [52]. Near the end period of the scoping review, several studies were published discussing the transformation of routine hospital appointments for pregnant women and cancer patients (priority groups) in order to mitigate nosocomial infections [53–57].

### Social impacts

Articles were examined for mention of the economic (i.e. loss of income) and social (i.e. family violence) impacts of COVID-19. Social impact studies tended to especially focus on the mental health of healthcare workers, students, and the general population (i.e. sleep quality, stress, anxiety) during lockdown (n: 126 in Chinese and English). In other words, the focus is on how these populations were managing or would manage the return to “normality” after the lockdown. The vast majority of papers in particular are concerned with healthcare workers’ mental health and their return to “normal” life after the outbreak. As noted above, the majority of healthcare workers responding to COVID-19 were women (three quarters of the healthcare workforce in China are women [9]).

One study documented Post-Traumatic Stress Syndrome (PTSS) amongst the healthcare workers who worked in the COVID-19 outbreak hospitals [58]. In one study, being male was identified as a “protective” factor for depression among doctors [59]. This study “confirmed” the view that depression rates are universally higher in women, and that biological determinants, sex role changes, but also unspecified social factors might contribute to this difference [59]. One survey showed women experienced higher levels of psychological distress, and another study found women’s resilience was significantly lower than men [60, 61]. There was no examination in these studies of the social determinants, including gender norms, in seeking treatment and counselling for depression and other mental health conditions within China. Workplace roles, duties, and expectations of healthcare workers were not examined in these papers. There was no examination of the toll of double burden of homecare roles and responsibilities whilst working in a high-risk environment during a pandemic outbreak. For example, only two studies examined whether concerns with family infection—essentially bringing the virus home—was the biggest stress for medical staff [62–64].

Given the broadness of the social impact category it is striking how little knowledge was being circulated in real time about the economic and social impacts of the lockdown, and the burden of care roles and responsibilities amongst family units. Research published after this scoping review period has revealed some insights into the social and economic impacts of the Wuhan lockdown, especially on the unequal gendered experiences of this lockdown [18].^2^

### Security impacts

Finally, for this category we analysed articles that examined individual experience(s) of violence during the first wave, healthcare workers’ physical safety, and fear of transmission (within families and communities). Only a minority of studies published on the security impacts (*n* = 28) of COVID-19 in “real time” during the first wave. The majority of the security impact studies examined individuals’ fear of being in lockdown and fear of spreading the virus amongst family [65]. Despite real-time research in other first wave affected locations revealing domestic violence as an immediate consequence of lockdown measures, there was only one published paper documenting this experience in China [18]. Hongwei Bao documented the “Anti-domestic Violence Little Vaccine” campaign as a demonstration of how Chinese feminists engaged with the issues of domestic violence and women’s rights during the pandemic. This publication was the only one identified in the six-month period that studied individual experiences of violence during the first wave [66].

## Discussion

Our scoping research showed there was minimal analysis of gender differences in the “first wave” of published papers on the COVID-19 outbreak in China. Sex data was mostly used for clinical analysis and not gender analysis. From the analysis of Chinese and English published literature on the COVID-19 first wave in China there were two areas where sex-disaggregated data was utilised to examine groups’ experiences of COVID-19: female healthcare workers and pregnant women. Very few papers that published on these topics examined the gendered experiences amongst the populations affected by lockdown.

Research into the impact of COVID-19 on women healthcare workers’ mental health was viewed from a heteronormative gender lens where women were described as more prone to anxiety and stress [67]. The fact that the majority of healthcare workers were women, and traditional gendered expectations required women to still manage family responsibilities, was only mentioned once [64]. Most of the focus was on their anxiety, depression, post-traumatic stress symptoms (PTSS) and poor sleep quality, and women’s biological predisposition to stress.

Along with highlighting the particularly tough conditions which healthcare workers endured during the pandemic, several papers called for specific policy measures to protect and support them. The most common recommendation was to provide psychological support and interventions [68], such as health education and training, and to focus on the healthcare workers’ safety measures (i.e. provision of protective equipment) [69–78]. Individual level concerns about family welfare during their absence [77], individual power to challenge employment conditions [79–81], and physical challenges posed by the lockdown were ignored or side-lined in the majority of published research.

As the rate of infection of healthcare workers in Wuhan grew during the first wave, the proportion of women in infection cases also increased. There was a need to strategically encourage and mobilise healthcare workers between provinces and cities. This required research into training, mobilisation, infection control and, related to the above, addressing attendant mental health challenges that emerged with relocation, long hours, and quarantine. The literature, however, seemed to assume these challenges were due to women’s disposition to mental illness rather than the fact that women healthcare workers make up the majority of the healthcare workforce but would experience economic, social and security challenges unique to gendered roles and expectations within Chinese society [82, 83].

The scoping review found a common association between women healthcare workers and mental health. Stress and anxiety due to uncertainty, fear, and long working hours were attributed to women healthcare workers more than to their male counterparts. These findings appear to be more attributable to gender stereotypes.

Real-time research on gender determinants for infection, risk and vulnerability was minimal. This finding is consistent with other studies that have found gender research gaps in China’s response to other health epidemics including HIV/AIDS [84]. The only exception was pregnant women, but these studies were not gender studies of their experiences and stereotypes encountered during COVID-19 lockdown. The studies focused on pregnant women as a priority group as they need to access hospital facilities regularly and are at higher risk of infection in hospital. Coupled with this is a (gendered) cultural tradition that emphasises the importance of “mother and child” with most families still having low fertility rates. Protecting the health concerns and needs of expecting mums and the unborn is a very high priority in China [19].

There may be practical factors at play that determined the volume and thematic focus of the outputs: the publication process is shorter for public health and medical academic articles compared to social science journals (which tend to be the primary location for gender publications). Research about gender issues requires different methods of data collection and analysis which may be lacking amongst the disciplines publishing rapid research in the first stages of a health emergency. The published research focused on sex disaggregation to explain experiences of COVID-19 infection and lockdown impacts. It is important sex-disaggregated data is available in real time but this data alone is not sufficient for rapid gender analysis. The next step is to promote rapid research that can understand how gender drives behaviours, expectations, and resilience during an outbreak [85].

## Conclusion

At the outset of a health emergency, rapid research needs to pay attention to the gendered roles attached to infection control, healthcare access, risk interventions and social welfare.

In China, nationwide collection of sex-disaggregated data was not initially prioritised at the onset of the outbreak. This was not unique to China. The sex-disaggregated research published during the first wave revealed high rates of infection amongst healthcare workers, the majority of them being women. Women healthcare workers’ mental stress may have had nothing to do with their “biological tendencies” but their real risk of exposure. The exclusion of this knowledge in real time affects the design of effective prevention and recovery. There was very little research on the social, security, and economic drivers of the pandemic during the first wave of COVID-19 in China. The knowledge gaps that occur in the first wave of an outbreak may be tied to the research and policy directives prioritised at the start of the pandemic and in the recovery stages. Capturing sex-disaggregated data is the first step. The next step is to examine the how gender stereotypes and gendered differences lead to different patterns of exposure and treatment in China. There needs to be a research-policy feedback loop that values this research to ensure the design of even more effective policy. Gender analysis during the first stage of an outbreak can assist with evaluation of most effective public health measures, identify access to health barriers amongst priority communities, and serve to create a feedback loop for more effective gender-inclusive policy and recovery.

## Supplementary Information

**Additional file 1.** Keywords and search syntax.

## Data Availability

Datasets are available through the corresponding author upon request.

## Abbreviations

HCWs: Healthcare Workers
PRISMA: Preferred Reporting Items for Systematic Reviews and Meta-Analysis
TCM: Traditional Chinese Medicine
IAEG-SDGs: Inter-Agency and Expert Group on Sustainable Development Goals Indicators

## Footnotes

1 For the Google search, the authors reviewed the first 200 results yielded sorted by relevance. For the Chinese literature, the authors selected studies published in the Guide to the Core Journals of China 2019 (version 8). The guide provides comprehensive comments on the quality and level of academic influence of Chinese periodicals. Only accepted and published studies were included in this study.

2 A comparison of different countries analysed for gendered impacts of the COVID-19 outbreak may be viewed on the Gender and COVID-19 Matrix. https://www.genderandCOVID-19.org/matrix/ (Access 20 October 2021).
