# Supplementary material for: COVID-19, sex, and gender in China: a scoping review
Source: Global Health. 2022 Feb 4;18:9. doi: 10.1186/s12992-022-00804-w (PMC8815015; doi:10.1186/s12992-022-00804-w)
Supplement: Supplementary file 1 — Additional file 1. Keywords and search syntax. [file 12992_2022_804_MOESM1_ESM.docx]

**Supplement A**

TS=("Covid-19" OR "Sars-cov-2" OR "new coronavirus pneumonia" OR "NCP" OR "coronavirus" OR "Wuhan pneumonia" OR "Wuhan new pneumonia" OR "Wuhan new coronary pneumonia" OR "SARS-like" OR "SARS-Cov-2" OR "infectious disease*" OR "epidemic" OR "outbreak*" OR "pandemic" OR "Public health emergency*") AND TS=("gender" OR "sex*" OR "women" OR "girl*" OR "men" OR “boy”) **Refined by:** **LANGUAGES:** (ENGLISH) AND **COUNTRIES/REGIONS:** (PEOPLES R CHINA) AND **DATE**: (Jan 2020 – June 2020)

主题:(新型冠状病毒肺炎+新冠肺炎+新冠病毒+武汉肺炎+武汉新型肺炎+武汉新冠肺炎+疑似SARS+NCP+武汉病毒+传染病+疫情+防疫)

主题:(女性+女 +妇女+性别+男性+男)
